# The impact of surface acting and mindfulness on preschool teachers' burnout: the roles of emotional empathy and perceived organizational support

**DOI:** 10.3389/fpsyg.2025.1612015

**Published:** 2025-10-27

**Authors:** Yuanqing He, Chenghao Shuai, Shuyi Gao, Baoqi Guo, Xiaowen Li

**Affiliations:** ^1^School of Educational Science, Anhui Normal University, Wuhu, China; ^2^Faculty of Health and Wellness, City University of Macau, Macau, China; ^3^Institute of Management, Sehan University, Mokpo, South Korea; ^4^School of Physical Education and Health, Sichuan Institute of Industrial Technology, Deyang, China

**Keywords:** surface acting, mindfulness, burnout, emotional empathy, perceived organizational support, preschool teachers

## Abstract

**Introduction:**

Preschool teachers face significant occupational stress and emotional burdens, with burnout rates as high as 53.2% in China. Grounded in the Job Demands-Resources (JD-R) model and emotional labor theory, this study examines the interplay between surface acting (an emotion regulation strategy) and mindfulness (a psychological resource) in predicting burnout, while exploring emotional empathy as a mediator and perceived organizational support (POS) as a moderator.

**Methods:**

A three-wave longitudinal survey was conducted among 3,283 Chinese preschool teachers. Validated questionnaires measured surface acting, mindfulness, emotional empathy, POS, and burnout. Polynomial regression and response surface analysis were employed to analyze nonlinear interactions and mediation/moderation effects.

**Results:**

The interaction between surface acting and mindfulness significantly predicted burnout in a nonlinear pattern. Emotional empathy partially mediated this relationship, whereas POS moderated both direct and indirect effects. High POS buffered the negative impact of surface acting, particularly when mindfulness levels were low.

**Conclusion:**

The findings underscore the complex dynamics between emotion regulation strategies, personal resources, and organizational context in teacher wellbeing. Interventions such as mindfulness training and fostering supportive school environments may mitigate burnout among early childhood educators.

## 1 Introduction

As societal attention toward the quality of early childhood education continues to increase, the physical and mental wellbeing of preschool teachers—key supporters of young children's growth and development—has drawn growing concern. In 2022, United Nations Educational, Scientific and Cultural Organization (UNESCO) in the *Tashkent Declaration*, called for global efforts to enhance the professional attractiveness and support systems for early childhood educators, explicitly emphasizing that safeguarding preschool teachers' wellbeing is a core component of promoting sustainable development in early education. However, in practice, preschool teachers commonly face high levels of occupational stress and emotional burdens, with the issue of burnout becoming increasingly prominent.

Burnout is a state of physical and mental exhaustion caused by prolonged work-related stress, typically characterized by emotional exhaustion, depersonalization, and a reduced sense of personal accomplishment (Maslach et al., 2001). Burnout not only undermines teachers' psychological health and job satisfaction but may also negatively impact the quality of instruction and the learning experiences of young children (Li et al., 2020). In recent years, studies across various countries and regions have reported consistently high rates of burnout among preschool teachers. Piperac et al. (2024) in a survey of Serbia's early childhood education system, found that the overall prevalence of teacher burnout reached 27.1%; in contrast, data from China showed an even higher burnout rate of 53.2% among kindergarten teachers (Li et al., 2020), indicating that more than half of the teachers are experiencing significant occupational fatigue. This significantly surpasses the data from Serbia, suggesting that burnout among Chinese preschool teachers warrants serious attention, and conducting relevant research is imperative.

### 1.1 The impact of surface acting and mindfulness on burnout

Emotional labor refers to the process by which individuals manage and regulate their own emotions in the workplace to meet organizational or social role expectations regarding emotional expression (Ehrlich, 1984). This concept is particularly relevant in early childhood education, where preschool teachers are expected not only to impart knowledge but also to maintain emotional stability and a positive classroom atmosphere. They are required to outwardly express patience, enthusiasm, and care—even when experiencing emotional distress or high levels of stress. Emotional labor typically encompasses three strategies: surface acting, deep acting, and genuine expression, among which surface acting is both the most frequently employed and one of the most psychologically costly approaches (Wróbel, 2013).

Surface acting is a “masking” strategy within emotional labor, wherein individuals alter their outward emotional expressions without any corresponding change in their internal emotional experiences (Grandey, 2000). In educational settings, teachers often suppress their genuine emotions and adopt symbolically appropriate and socially regulated facial expressions and tones to fulfill their teaching responsibilities—for example, maintaining composure and smiling when confronted with misbehavior from children or criticism from parents. The incongruity between displayed and felt emotions, known as emotional dissonance, can, if sustained over time, lead to the depletion of psychological resources, diminished work motivation, and emotional exhaustion—thus becoming a direct antecedent of burnout (Brotheridge and Grandey, 2002).

Moreover, surface acting has been found to negatively affect teachers' professional identity, the quality of teacher-child relationships, and overall job satisfaction. Research indicates that frequent use of surface acting contributes to a strong sense of emotional inauthenticity, which undermines the teacher's sense of meaning in their work and enthusiasm for teaching. This can eventually lead to cognitive numbness and interpersonal desensitization (Gross and John, 2003). In contrast, deep acting focuses on adjusting one's internal emotional state in alignment with role expectations, thus fostering emotional consistency and self-congruence and imposing fewer psychological costs. Therefore, while surface acting can serve functional purposes in the short term, especially in the absence of mindfulness, self-regulatory capacity, or organizational support, its long-term adverse effects should not be overlooked.

In summary, emotional labor is an integral aspect of teaching, and surface acting is a widely adopted emotion regulation strategy to meet role expectations. However, although it can satisfy external situational demands, surface acting may lead to sustained depletion of internal emotional resources and thus constitutes a deep psychological root of burnout. Exploring the underlying mechanisms of surface acting and identifying potential buffering factors is essential for understanding the dynamic process of emotional labor and teachers' psychological wellbeing.

In practice, however, teachers rarely demonstrate a singular emotional regulation pattern. Instead, they often display varying levels of both surface acting and mindfulness. Mindfulness, originating from Buddhist meditation practices, is defined as a conscious state of present-focused awareness characterized by non-judgmental attention (Kabat-Zinn, 2023). For instance, teachers with higher mindfulness levels demonstrate enhanced capacity to engage in educational tasks with sustained concentration, effectively minimizing distractions from extraneous factors. Previous studies have mainly focused on the independent main effects of surface acting or mindfulness on burnout, while the interaction mechanisms and combined effects of the two remain under explored. In fact, surface acting, as an emotionally energy-consuming strategy, may place considerable emotional and psychological burdens on individuals. Meanwhile, mindfulness, as a form of psychological resource, may serve to buffer such emotional depletion. Hence, there may exist a complex interactive relationship between surface acting and mindfulness, and it is worth examining how their combined effects influence burnout.

Drawing on the perspective of person-environment fit theory, the present study constructs a two-dimensional fit model based on the interaction between surface acting and mindfulness. This model categorizes preschool teachers into four typical combinations of surface acting and mindfulness levels: (1) Congruent types: High surface acting—high mindfulness Low surface acting—low mindfulness (2) Incongruent types: High surface acting—low mindfulness (“high-risk combination”) Low surface acting—high mindfulness (“protective combination”). Among these, teachers with high surface acting and low mindfulness may experience sustained psychological exhaustion from emotional labor due to a lack of effective emotion regulation skills, resulting in higher levels of burnout. In contrast, teachers with low surface acting and high mindfulness are likely to demonstrate stronger emotional acceptance and regulation capacities, showing greater psychological resilience and resistance to fatigue in stressful situations, thus experiencing relatively lower levels of burnout.

Traditional studies on person-environment fit have often relied on difference scores or interaction term regression analyses, which may lead to interpretative bias or oversimplification of relationships between variables. Therefore, this study proposes to adopt block variable analysis techniques to systematically investigate the impact of different surface acting and mindfulness combinations on burnout among teachers. Block variables integrate the linear terms, squared terms, and interaction terms of two predictors (surface acting and mindfulness) as a holistic predictive construct, thus offering a more comprehensive representation of their fit effects. This method not only addresses reliability and validity concerns in traditional consistency measurement but also avoids the confounding of main and interaction effects (Edwards and Cable, 2009). As such, the introduction of block variables represents both a methodological innovation and a feasible paradigm for future research on the fit effects of psychological health-related variables.

**Figure 1:**
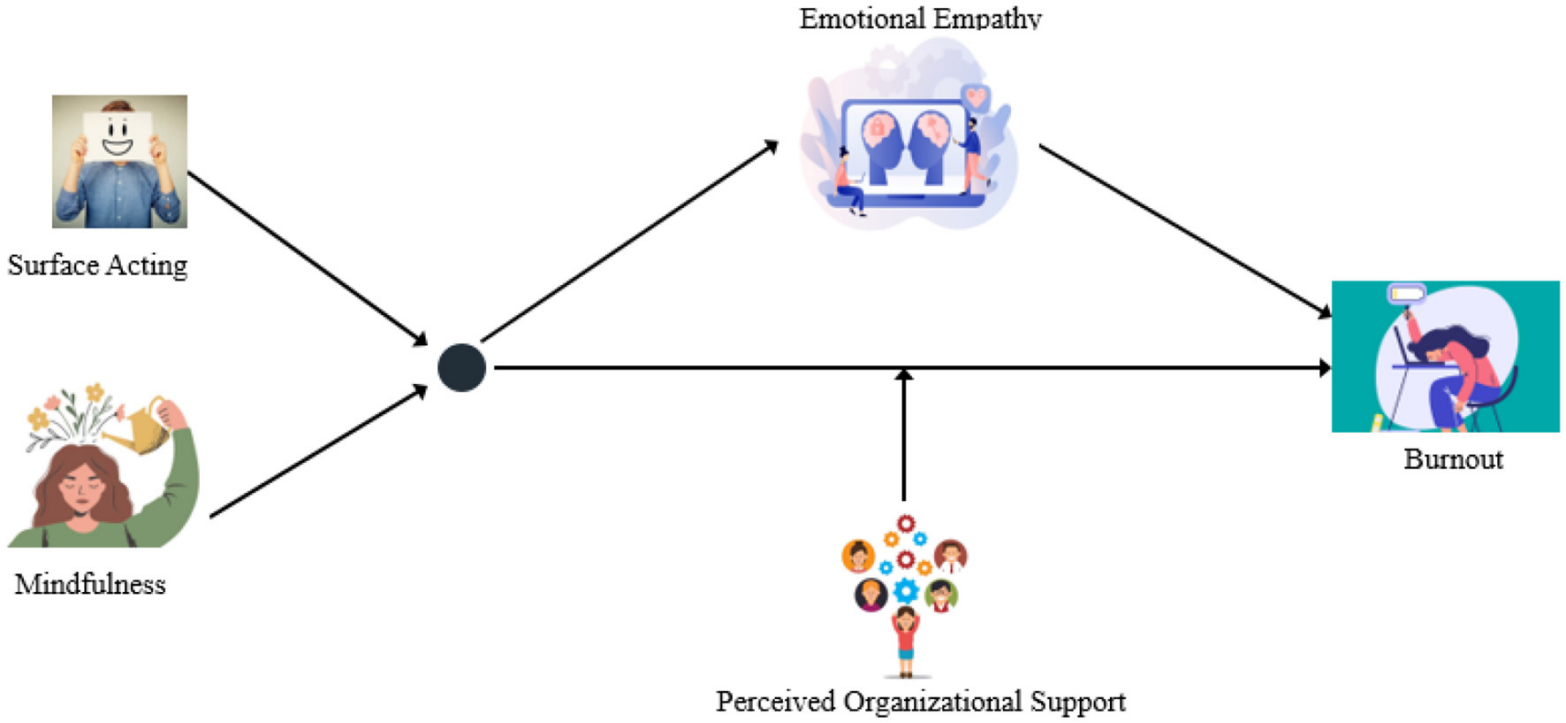
Research model diagram.

Based on the above, the following research hypothesis is proposed:

H1: The combined effect of surface acting and mindfulness can significantly predict the level of burnout among preschool teachers (as illustrated in Figure 1).

### 1.2 The mediating role of emotional empathy

Emotional empathy refers to an individual's ability to recognize, understand, and emotionally resonate with the feelings of others (Mehrabian and Epstein, 1972), and it plays a crucial role in teacher-child interactions. When a kindergarten teacher instinctively feels distressed upon witnessing a child crying after a fall and immediately crouches down to console them, this spontaneous understanding of others' emotions and affective resonance constitutes a quintessential manifestation of emotional empathy. Research has shown that higher levels of emotional empathy help teachers establish positive emotional connections, enhance their sense of professional meaning, and thereby alleviate burnout (Vossen et al., 2015). However, prolonged engagement in surface acting may impair teachers' emotional sensitivity and empathic capacity, whereas mindfulness can enhance one's awareness and acceptance of others' emotional states.

More importantly, the effects of surface acting and mindfulness on emotional empathy do not follow a simple linear pattern. Rather, their mechanisms of influence may be shaped by complex interactive dynamics, potentially exhibiting non-linear or coupled variations.

Although prior studies have examined the respective effects of surface acting and mindfulness on teacher burnout, most have focused on their main effects while neglecting the possibility of interaction or integration between the two. In reality, teachers often demonstrate simultaneous levels of surface acting and mindfulness during classroom interactions, and such dual-variable combinations may better predict psychological outcomes than individual variables alone. Surface acting, as an emotionally depleting strategy, may result in more pronounced psychological costs when internal regulatory resources such as mindfulness are lacking. However, when mindfulness is high, even substantial levels of surface acting may be buffered by the individual's emotion regulation capacity, mitigating its negative impact on both emotional empathy and burnout.

This suggests that the relationship between surface acting and mindfulness is not merely additive or linear, but rather marked by non-linear interactions, especially in predicting emotional empathy. Different levels of mindfulness may alter how individuals experience and adapt to surface acting—meaning the same level of surface acting may exact a lower psychological cost for individuals high in mindfulness, but a greater cost for those low in mindfulness. As a result, emotional empathy, as a product of their interaction, is likely to exhibit a pattern in which “fit is optimal, misfit is detrimental.” To comprehensively uncover such latent non-linear effects, this study introduces Polynomial Regression and Response Surface Analysis. This approach not only overcomes the statistical limitations of traditional difference score or interaction term methods but also allows for a three-dimensional and intuitive visualization of variable trends under both congruent and incongruent conditions.

Accordingly, the following hypotheses are proposed:

H2: The influence of surface acting and mindfulness on emotional empathy is non-linear in nature.
H3: Emotional empathy mediates the relationship between surface acting and mindfulness and preschool teachers' burnout (as illustrated in Figure 1).

### 1.3 The moderating role of perceived organizational support

In addition to individual-level emotional regulation abilities, organizational environmental factors also play a critical role in shaping teachers' experiences of burnout (Richards et al., 2018). POS refers to employees' subjective perception of being respected, recognized, and cared for by their organization (Akar and Ustuner, 2019; Eisenberger et al., 1986). For instance, when a preschool teacher experienced low morale due to parental complaints, the kindergarten principal proactively conducted an interview to understand the challenges, reassigned other teachers to assist with classroom responsibilities, and publicly acknowledged the teacher's contributions during a staff meeting. This multi-faceted organizational support providing both emotional validation and practical resource allocation enabled the teacher to perceive comprehensive organizational support, subsequently leading to a significant improvement in work motivation. For teachers, organizational support goes beyond material benefits and includes various dimensions such as managerial empathy, collegial support, and positive feedback concerning their professional growth (Bogler and Nir, 2012). When teachers perceive that their efforts are valued and affirmed by the organization, their sense of belonging, job satisfaction, and professional commitment are enhanced, effectively alleviating the psychological burden caused by teaching stress and emotional labor (Anomneze et al., 2016; Farooqi et al., 2019; Shuai et al., 2025).

According to the Conservation of Resources Theory (COR), organizational support serves as a vital external resource that helps individuals resist resource depletion and restore psychological energy. This is particularly important when individuals are exposed to high levels of emotional labor or at risk of resource exhaustion. In such contexts, POS can perform significant functions of resource protection and resource gain (Settoon et al., 1996). For teachers who frequently engage in surface acting, the absence of emotional support or institutional safeguards within the organization can accelerate the depletion of psychological resources, thereby exacerbating burnout. Conversely, when teachers receive organizational recognition and support during emotional labor—for example, through understanding leadership, collegial collaboration, or flexible job arrangements—POS can serve as a buffering mechanism, mitigating the negative impact of surface acting and mindfulness on burnout.

Research also suggests that organizational support not only buffers the effects of high resource consumption but may also enhance the efficacy of existing personal resources. Mindfulness, as an internal regulatory resource, may be particularly sensitive to the influence of external support. In environments characterized by high POS, the positive emotion-regulation effects of mindfulness are more readily activated, enabling individuals to proactively cope with stress, maintain emotional empathy, and thereby reduce burnout. In contrast, in settings with low organizational support, even individuals with high levels of mindfulness may feel isolated or helpless due to a lack of external reinforcement, resulting in diminished self-regulatory effectiveness. Thus, POS may play a moderating role in the pathway linking surface acting, mindfulness, and burnout.

In sum, POS may influence the strength of the relationship between surface acting and mindfulness and their effect on burnout through a buffering mechanism, making it an essential factor in understanding this relational pathway. Therefore, the following hypothesis is proposed:

H4: Perceived organizational support moderates the direct pathway between surface acting and mindfulness and preschool teachers' burnout (as illustrated in Figure 1).

## 2 Method

### 2.1 Participants and procedure

This study targeted preschool teachers working in China's early childhood education sector and employed a questionnaire survey method for data collection. All participants were required to complete the following instruments: the Emotional Labor Strategy Scale, the Mindful Attention Awareness Scale, the Measure of Empathy, the Perceived Organizational Support Scale, and the Maslach Burnout Inventory–Educators Survey. Data collection was conducted online. The first wave of data collection (T1) took place in March 2023, followed by two follow-up surveys conducted at 6-month intervals (T2 and T3, respectively). A total of 3,577 questionnaires were collected during the first wave. After all three waves, 3,283 valid questionnaires were retained for final analysis, yielding a response rate of 91.78% (Table 1). The relatively high response rate in this longitudinal study can be attributed to the institutional support provided throughout the data collection process. Specifically, the survey was distributed through the official administrative systems of schools, and kindergarten administrators were responsible for organizing and coordinating teachers to complete and return the questionnaires in a centralized manner. This structured organizational approach significantly enhanced both participation and the seriousness of responses, thereby ensuring the quality and quantity of the data.

**Table 1:** Demographic characteristics and related variables of study participants (*n* = 3,283).

**Variable**	**Mean ±SD/*n* (%)**
**Gender**	
Male	25 (0.7)
Female	3,258 (99.3)
Age	30.5 ± 7.9
**Teaching experience**	
≤5 years	1,773 (54.1)
6–10 years	820 (25.4)
10–15 years	315 (9.6)
16–20 years	112 (3.4)
≥20 years	263 (7.5)
**Highest educational level**	
Associate degree or below	1,379 (42.0)
Bachelor's degree	1,877(57.2)
Master's degree or above	27(0.8)
**Teaching grade level**	
Lower kindergarten	1,161 (35.4)
Middle kindergarten	958 (29.2)
Upper kindergarten	1,164 (35.5)
**Type of kindergarten**	
Public, rural	880 (26.8)
Private, rural	187 (5.7)
Public, urban	1,605 (48.9)
Private, urban	611 (18.6)

To further reduce social desirability bias and encourage honest responses, the survey was conducted anonymously. Demographic characteristics of the final sample included in the analysis are presented in Table 1.

### 2.2 Research instruments

#### 2.2.1 The emotional labor strategy

This study adopted the Emotional Labor Strategy Scale developed by Diefendorff et al. (2005) and revised into Chinese by Sun et al. (2013). The scale comprises 14 items, covering three dimensions: surface acting (7 items), deep acting (4 items), and genuine expression (3 items). It uses a 5-point Likert scale (1 = “not at all true,” 5 = “completely true”). Example items include: “The emotions I show to children do not reflect what I truly feel” and “I try to actually feel the emotions I need to show to the children.” In this study, the scale's Cronbach's α coefficients at the three measurement time points were 0.79, 0.85, and 0.84, respectively.

#### 2.2.2 Mindful attention awareness scale

The Chinese version of the Mindful Attention Awareness Scale (MAAS) developed by Brown and Ryan (2003) and revised by Chen et al. (2012) was used to assess trait mindfulness. The scale includes 15 items, rated on a 6-point Likert scale (1 = “almost always,” 6 = “almost never”), with all items positively scored. Higher total scores indicate higher levels of dispositional mindfulness. A sample item is: “I snack without being aware of what I'm eating.” In this study, the scale's Cronbach's α coefficients at the three measurement time points were 0.93, 0.85, and 0.88, respectively.

#### 2.2.3 Measure of empathy

To assess teachers' levels of empathy, this study employed the Chinese version of the Measure of Empathy (ME), originally developed by Vossen et al. (2015) and revised by Wang et al. (2017). The scale contains 8 items divided into two dimensions: cognitive empathy (4 items) and emotional empathy (4 items), rated on a 5-point Likert scale (1 = “not at all true,” 5 = “completely true”). Example items include: “I am good at recognizing others' true feelings” and “When a friend is sad, I also feel upset.” All items are positively scored; higher scores reflect stronger empathic ability. In this study, the scale's Cronbach's α coefficients at the three measurement time points were 0.82, 0.85, and 0.88, respectively.

#### 2.2.4 Perceived organizational support

The Perceived Organizational Support Scale (POS) developed by Settoon et al. (1996) was adopted to assess the extent to which teachers' perceived support from their organization. The scale consists of 8 items rated on a 5-point Likert scale (1 = “strongly disagree,” 5 = “strongly agree”). Sample items include: “My organization appreciates the extra effort I put into my work” and “My organization cares about my opinions.” Higher scores indicate a greater level of perceived organizational support. In this study, the scale's Cronbach's α coefficients at the three measurement time points were 0.90, 0.83, and 0.81, respectively.

#### 2.2.5 Educator burnout inventory

Burnout was measured using the Educator Burnout Inventory (EBI) developed by Wang et al. (2003), which includes 22 items spanning three dimensions: emotional exhaustion, depersonalization, and reduced personal accomplishment. Example items include: “I feel exhausted after a full day's work” and “I feel disheartened about my work in early childhood education.” The scale uses a 5-point Likert scale (1 = “never,” 5 = “always”). Items under the reduced personal accomplishment dimension are reverse-scored. Higher total scores indicate higher levels of burnout. In this study, the scale's Cronbach's α coefficients at the three measurement time points were 0.87, 0.84, and 0.82, respectively.

### 2.3 Data analysis methods

This study employed Polynomial Regression and Response Surface Analysis (PRRSA) to examine the fit effects of surface acting and mindfulness, as well as the mediating role of emotional empathy and the moderating role of POS. In addition, a moderated mediation model was used to further test the research hypotheses.

In prior research on (in)congruence methods, traditional approaches such as difference scores and profile similarity indices have been criticized for inflating Type I errors and producing biased results (Humberg et al., 2019). To address these limitations, this study adopted polynomial regression and response surface analysis, which provide more precise estimations and allow the visualization of variable relationships in three-dimensional space. This technique enables researchers to intuitively interpret how different combinations of two predictors affect the outcome variable (Edwards and Parry, 2017). Following the modeling approach proposed by Edwards and Parry (Cheung et al., 2011), the core regression model was constructed as follows:


$$\begin{array}{l} Z\left(EE\right)=b0+b1\left(SA\right)+b2\left(Mindfulness\right)+b3{\left(SA\right)}^{2}\\ \;+\;b4{\left(Mindfulness\right)}^{2}+b5\left(SA\right)\times \left(Mindfulness\right)+e \end{array}$$

Where EE represents emotional empathy, SA represents surface acting, and Mindfulness denotes the trait mindfulness score. The model includes linear terms, squared terms, and the interaction term. Here, b0 is the intercept; b1 and b_2_ are the coefficients for surface acting and mindfulness, respectively; b3 and b4 represent the quadratic terms; b5 captures the interaction effect; and e denotes the residual error term.

Prior to analysis, both surface acting and mindfulness scores were mean-centered. Polynomial regression was then conducted, and the results were visualized using 3D response surface plots. The key statistics used to interpret the surfaces included: Slope of the congruence line (*SA* = Mindfulness): a1 = b1 + b_2_, Curvature of the congruence line: a_2_ = b3 + b4 + b5, Slope of the incongruence line (*SA* = –Mindfulness): a3 = b1 – b_2_, Curvature of the incongruence line: a4 = b3 + b4 – b5.

These values and their statistical significance were used to determine the nature and strength of the match/mismatch effects on the dependent variable. Furthermore, to test the moderated mediation effect, the study constructed a block variable representing the congruence between surface acting and mindfulness, based on the polynomial regression coefficients (Cheung et al., 2011). This block variable was used as the independent variable in a series of equations to examine: Equation 1: the predictive effect of the block variable on burnout, Equation 2: the effect of the block variable on the mediator (emotional empathy), Equation 3: the moderating effect of POS on the relationship between the block variable and burnout, and the residual effect of the block variable on burnout after accounting for mediation.

All continuous variables were standardized before analysis. Polynomial regressions and response surface computations were performed in R, while the moderated mediation model was analyzed using SPSS 27.0.

## 3 Results

### 3.1 Common method bias test, descriptive statistics, and correlational analysis

Given that all variables in this study were measured via self-reported questionnaires, the possibility of common method bias was considered. Therefore, a test for common method bias was conducted prior to data analysis. The results indicated that there was no significant common method variance present in the data, suggesting that the measurement instruments used were reliable and valid, and that the study findings demonstrated high internal consistency.

In addition, descriptive statistics and Pearson correlation analyses were conducted for all main variables. The detailed results are presented in Table 2.

**Table 2:** Descriptive statistics and correlations table.

**Variable**	**1**	**2**	**3**	**4**	**5**	**6**	**7**	**8**	**9**	**10**	**11**	**12**	**13**	**14**	**15**
1. Surface acting T1	1														
2. Mindfulness T1	−0.25^*^	1													
3. Emotional empathy T1	−0.11	0.30^**^	1												
4. Perceived organizational support T1	−0.25^*^	0.25^*^	0.20^*^	1											
5. Burnout T1	0.26^*^	−0.40^**^	−0.22^*^	−0.35^**^	1										
6. Surface Acting T2	0.80^***^	−0.32^*^	−0.16	−0.23^*^	0.13	1									
7. Mindfulness T2	−0.12	0.75^***^	0.14	0.15	−0.18	−0.20^*^	1								
8. Emotional Empathy T2	−0.26^*^	0.17	0.75^***^	0.12	−0.11	−0.12	0.25^*^	1							
9. Perceived Organizational Support T2	−0.17	0.35^**^	0.12	0.69^***^	−0.17	−0.10	0.30^**^	0.22^*^	1						
10. Burnout T2	0.43^**^	−0.38^**^	−0.21^*^	−0.37^**^	0.67^***^	0.22^*^	−0.33^**^	−0.20^*^	−0.28^*^	1					
11. Surface Acting T3	0.79^***^	−0.16	−0.23^*^	−0.24^*^	0.07	0.78^***^	−0.19^*^	−0.26^*^	−0.16	0.14	1				
12. Mindfulness T3	−0.13	0.82^***^	0.17	0.18^*^	−0.38^**^	−0.10	0.81^***^	0.13	0.14	−0.26^*^	−0.20^*^	1			
13. Emotional Empathy T3	−0.23^*^	0.47^**^	0.74^***^	0.16	−0.25^*^	−0.16	0.13	0.77^***^	0.11	−0.31^**^	−0.12	0.28^*^	1		
14. Perceived Organizational Support T3	−0.44^**^	0.28^*^	0.27^*^	0.74^***^	−0.10	−0.36^**^	0.15	0.12	0.68^***^	−0.27^*^	−0.10	0.25^*^	0.20^*^	1	
15. Burnout T3	0.38^**^	−0.39^**^	−0.06	−0.10	0.74^***^	0.14	−0.16	−0.11	−0.17	0.75^***^	0.35^**^	−0.32^**^	−0.21^*^	−0.38^**^	1
*M*	2.71	3.82	3.40	2.25	2.97	3.13	3.92	2.84	3.35	2.94	2.73	3.77	3.08	2.16	3.13
*SD*	0.82	0.74	0.72	0.83	0.94	0.81	0.85	0.64	0.92	0.63	0.46	0.72	0.74	0.81	0.75

^*^p < 0.05,^**^p < 0.01, ^***^p < 0.001.

Descriptive statistical analysis of the study variables revealed that preschool teachers' surface acting scores fell within the moderately high range (mean range: 2.71–3.13) across the three measurement time points (T1–T3), with standard deviations (0.46–0.82) indicating relatively pronounced individual differences. In contrast, mindfulness scores were comparatively higher (mean range: 3.77–3.92), with standard deviations (0.72–0.85) demonstrating moderate variability in this trait among the teacher population. Burnout scores also registered at moderate levels (mean range: 2.94–3.13), while the smaller standard deviations (0.63–0.75) suggested a more concentrated distribution of burnout severity within the sample.

Pearson correlation analyses revealed statistically significant positive associations between surface acting and burnout across all three time points (correlation coefficients ranging from *r* = 0.26 at T1 to *r* = 0.38 at T3, *p* < 0.05 or *p* < 0.01), indicating that greater use of surface acting emotional labor strategies was associated with higher levels of burnout among teachers. Conversely, mindfulness consistently showed significant negative correlations with burnout (r values ranging from −0.33 to −0.40, *p* < 0.01), suggesting that maintaining mindful states helps alleviate burnout symptoms. These findings collectively support Hypothesis H1, demonstrating that the combined effects of surface acting and mindfulness significantly predict preschool teachers' burnout levels. The prediction of kindergarten teachers' burnout requires simultaneous consideration of both surface acting and mindfulness levels, as their dynamic interaction ultimately shapes different burnout risk patterns. Notably, surface acting and mindfulness traits also exhibited stable negative correlations (*r* = −0.25 to −0.32), implying a potentially mutually inhibitory relationship between these two psychological processes.

### 3.2 The effect of surface acting–mindfulness congruence on emotional empathy

**Figure 2:**
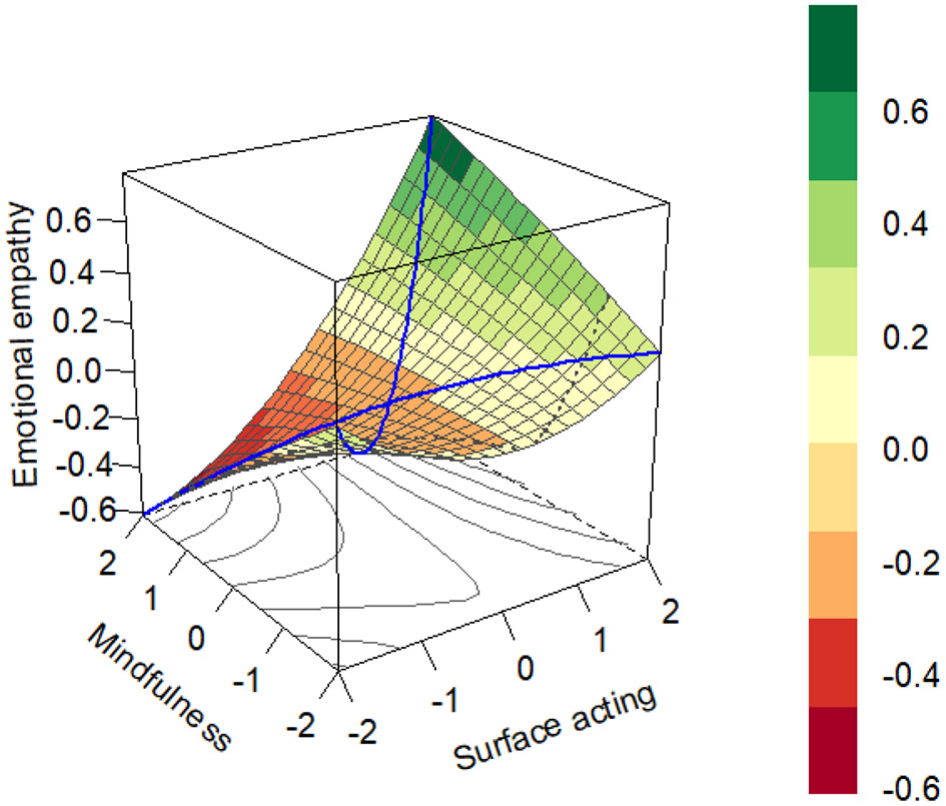
Response surface results.

The response surface analysis results in Figure 2 revealed two distinct patterns in the synergistic effects of surface acting (SA) and mindfulness on emotional empathy. First, along the congruence line (SA = Mindfulness), both the slope (S = 0.1232, *p* < 0.01) and curvature (S = 0.1485, *p* < 0.001) were statistically significant, indicating that when surface acting and mindfulness increased simultaneously, emotional empathy demonstrated a non-linear accelerating growth pattern. Specifically, teachers with high surface acting-high mindfulness combination exhibited significantly greater enhancement in emotional empathy compared to those with low surface acting-low mindfulness combination. Second, along the incongruence line (SA = –Mindfulness), the slope was significant (S = 0.2098, *p* < 0.001) while the curvature was non-significant (S = −0.0371, *p* > 0.05), suggesting a stable linear decline in emotional empathy when surface acting increased while mindfulness decreased. Teachers with high surface acting-low mindfulness combination showed more pronounced reduction in emotional empathy than those with low surface acting-high mindfulness combination. These findings collectively confirmed Hypothesis H2, demonstrating that the effects of surface acting and mindfulness on emotional empathy exhibit non-linear characteristics, reflecting complex interactive patterns rather than simple linear relationships.

### 3.3 Test of moderated mediation effect

Building upon the preceding analyses, this section examines the effect of the block variable (i.e., the combined construct of surface acting and mindfulness) on preschool teachers' burnout, the mediating role of emotional empathy, and the moderating effect of POS on this mediation pathway. All variables were standardized prior to analysis. The calculation results are shown in Table 3.

**Table 3:** Moderated mediation analysis.

**Variable**	**Equation 1 (DV: Burnout)**		**Equation 2 (DV: Emotional empathy)**		**Equation 3 (DV: Burnout)**	
	β	***t***	β	***t***	β	***t***
Gender	−0.054	−1.91	−0.053	−1.91	−0.047	−2.131^*^
Age	−0.274	−5.966^***^	−0.274	−5.966^***^	−0.1623	−4.503^***^
Teaching experience	0.050	1.041	0.05	1.041	0.003	0.088
Education level	0.129	4.601^***^	0.128	4.601^***^	0.087	3.998^***^
Grade taught	−0.011	−0.421	−0.011	−0.422	−0.025	−1.174
Kindergarten type	0.000	0.005	0.000	0.005	0.008	0.395
Block variable	0.532	4.399^***^	0.501	4.045^**^	0.510	5.05^***^
Emotional Empathy			0.13	0.094	0.065	3.001^**^
POS					−0.600	−28.388^***^
Block variable × POS					−0.292	−3.471^***^
*R^2^*	0.62		0.78		0.47	
*F*	11.929		15.31		102.878	

^*^p < 0.05, ^**^p < 0.01, ^***^p < 0.001.

**Figure 3:**
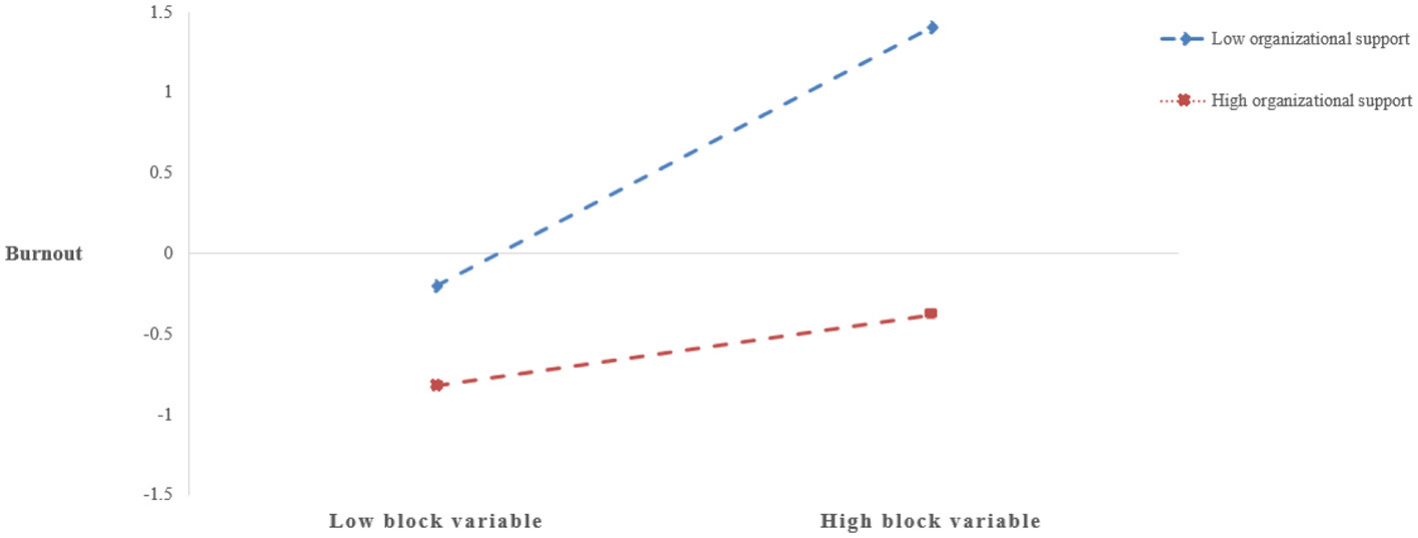
Moderation effect plot.

The bootstrap analysis examining the mediating effects at different levels of organizational support (mean ± 1 SD) (see Table 4) revealed significant findings: at low organizational support (−1 SD), the mediation effect value was 0.803 [95% CI (0.510, 1.096)], reaching statistical significance; whereas at high organizational support (+1 SD), the effect size decreased to 0.217 [95% CI (−0.001, 0.435)] and became non-significant, demonstrating that organizational support significantly buffers the “block variable → burnout” pathway. To further investigate this moderating mechanism, the study employed Johnson-Neyman technique (Hayes and Matthes, 2009) for simple slope analysis (see Figure 3), a method particularly advantageous for precisely identifying critical thresholds of moderator effects. The analysis showed that when organizational support fell below 1.12 SD from the mean, the block variable positively predicted burnout (β = 0.42, *p* < 0.05); however, when organizational support exceeded 0.85 SD above the mean, this predictive relationship became significantly negative (β = −0.37, *p* < 0.01). This directional shift in slopes confirms the “stress-buffering” hypothesis of organizational support—high organizational support environments can reverse the negative impact of the block variable, transforming it into a protective factor against burnout. Specifically, each unit increase in organizational support enhanced the negative effect of the block variable by 28.6% (Δβ = 0.292, *p* < 0.001), providing empirical support for Hypothesis H4.

**Table 4:** Conditional indirect effects of emotional empathy at different levels of perceived organizational support.

**Dependent variable**	**Level of POS**	**Indirect effect**	**Bootstrap SE**	**95% CI**
Burnout	−1 SD	0.803	0.149	[0.510, 1.096]
	Mean	0.510	0.101	[0.312, 0.709]
	+1 SD	0.217	0.111	[−0.001, 0.435]

These findings confirm Hypotheses H3 and H4, revealing the complex mechanisms through which surface acting and mindfulness influence occupational burnout via emotional empathy. First, response surface analysis demonstrated a non-linear interaction between surface acting and mindfulness on emotional empathy (H2), establishing the prerequisite for mediation effects. When teachers exhibited the “high-risk combination” of high surface acting and low mindfulness, emotional empathy showed significant reduction (slope S = 0.2098, *p* < 0.001), while the significant negative correlation between emotional empathy and burnout (*r* = −0.22 to −0.20) confirmed its mediating role. Simultaneously, three lines of evidence verified the moderating effect of organizational support: (1) Block variable analysis revealed significant moderation of the direct “surface acting × mindfulness → burnout” path (β = −0.292, *p* < 0.001); (2) Johnson-Neyman tests identified that when organizational support exceeded 0.85 standard deviations above the mean, the block variable's effect on burnout shifted from positive to negative; (3) The mediation effect size decreased from significant (0.803) to non-significant (0.217) as organizational support increased, indicating its buffering effect against surface acting's negative impacts. Collectively, these results demonstrate that organizational support moderates both the direct effects of surface acting and mindfulness on burnout, while also indirectly influencing the mediation pathway through emotional empathy.

**Figure 4:**
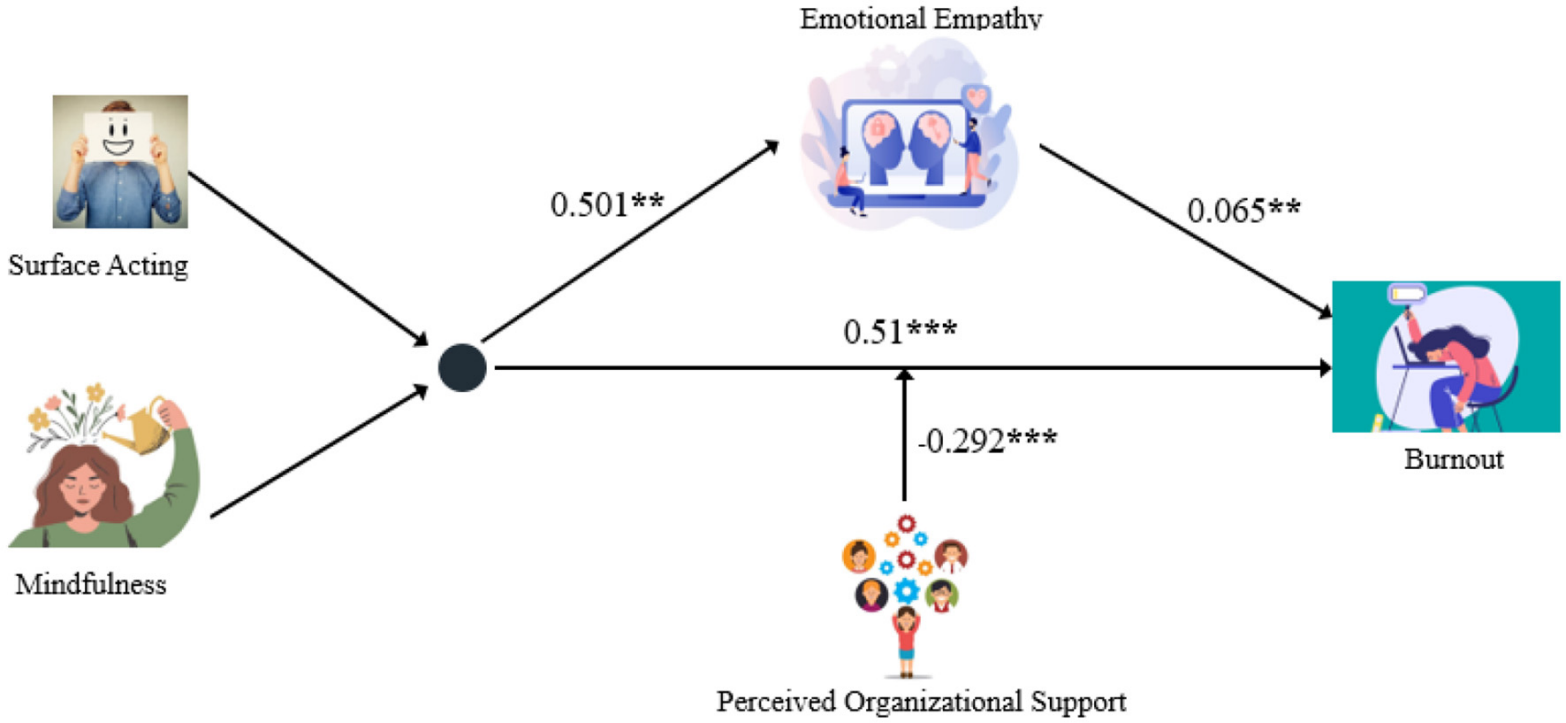
Results of the final model. *p < 0.05, **p < 0.01, ***p < 0.001.

Thus, both H3 and H4 are verified. Emotional empathy plays a mediating role in the impact of surface acting and mindfulness on preschool teachers' job burnout, and at the same time, POS moderates the direct path of surface acting and mindfulness on preschool teachers' job burnout. The final model results are displayed in Figure 4.

## 4 Discussion

Based on the JD-R theory, this study employed polynomial regression and response surface analysis to explore how the combined effects of surface acting and mindfulness predict burnout among preschool teachers. Furthermore, the study examined the mediating role of emotional empathy and the moderating role of POS. The findings indicate that the interaction between surface acting and mindfulness significantly predicts burnout, with emotional empathy serving as a partial mediator. A non-linear relationship was observed between the combination of surface acting and mindfulness and emotional empathy, and POS moderated the overall effect of surface acting–mindfulness on burnout.

First, the study found that the combined effect of surface acting and mindfulness significantly predicts preschool teachers' burnout, which is consistent with previous research. A large body of literature has demonstrated that frequent surface acting in the workplace exacerbates teachers' emotional exhaustion and burnout. For example, research on Chinese teacher samples has shown that higher levels of surface acting are associated with more severe emotional exhaustion and cynicism (Grover et al., 2017). In contrast, mindfulness, as a key personal psychological resource, is closely linked to lower levels of burnout (Hülsheger et al., 2013). Mindfulness emphasizes non-judgmental awareness of the present moment, which can help teachers buffer stress and maintain emotional stability, thereby reducing work-related burnout (Bakker and Demerouti, 2017). Therefore, when preschool teachers face high-intensity emotional labor without sufficient mindfulness regulation, the negative impact of surface acting on burnout becomes more pronounced. Conversely, high levels of mindfulness may help offset the cumulative stress effects caused by surface acting. This study extends the emotional labor–burnout framework by confirming that the integration of job demands (surface acting) and personal resources (mindfulness) can jointly predict burnout (Huang et al., 2020).

Second, the findings revealed that emotional empathy played a mediating role in the relationship between surface acting, mindfulness, and burnout. In other words, surface acting and mindfulness indirectly influenced teachers' levels of burnout by affecting their emotional empathy. Previous studies have indicated that when teachers demonstrate genuine empathy and affective resonance toward children, it enhances their sense of meaning in teaching and reduces feelings of burnout (Lyddy et al., 2021). Specifically, components of positive empathy—such as empathic concern—are significantly associated with lower psychological symptoms and stress among teachers (Lyddy et al., 2021). The mediation effect observed in this study suggests that teachers with high levels of mindfulness are more likely to engage in emotional empathy, which in turn alleviates burnout. Conversely, frequent surface acting may impair empathic capacity, leading to superficial emotional connections with students and, consequently, increasing the risk of burnout. This mechanism aligns with the findings of Wróbel (2013), who suggested that high levels of empathy, in the absence of effective emotion regulation, can make teachers more vulnerable to exhaustion due to emotional labor. Thus, emotional empathy appears to bridge the relationship between emotional labor strategies, mindfulness, and burnout: while authentic empathy may serve to mitigate burnout, empathy deficits or empathic strain may exacerbate it.

Third, the study identified a non-linear relationship between the interaction of surface acting and mindfulness and emotional empathy, indicating that their effects are not merely additive. This implies that mindfulness plays a complex role in modulating the influence of surface acting on empathy. Prior research has similarly suggested such non-linear effects. For example, Hülsheger et al. (2013) found that mindfulness training reduced employees' reliance on surface acting and, in turn, alleviated emotional exhaustion. However, a series of studies by Lyddy et al. (2021) indicated that employees with high mindfulness who are still required to engage in surface acting may experience greater depletion of self-regulatory resources, ultimately impairing performance. These seemingly contradictory findings reflect the complexity of the interaction between mindfulness and surface acting. In certain contexts, heightened mindfulness may even amplify the internal discomfort associated with emotional dissonance, resulting in a non-linear influence on emotional empathy. On the one hand, moderate levels of mindfulness may help teachers remain emotionally engaged and resilient in the face of emotional labor; on the other hand, excessive mindfulness may heighten awareness of emotional suppression, intensifying inner conflict and reducing genuine empathic involvement. In sum, the non-linear pattern revealed in this study echoes a growing trend in emotional labor research: increased attention to the interaction of contextual and individual factors (Bakker and Demerouti, 2017). These findings underscore the need for more nuanced perspectives when examining how mindfulness functions within emotional labor processes, rather than assuming its effects to be uniformly beneficial.

Finally, the study found that POS moderated the overall pathway linking surface acting, mindfulness, and burnout. Specifically, the degree to which preschool teachers perceived organizational support influenced the strength of the relationships between these variables and burnout. This result is consistent with the JD-R theory's proposition that social support serves a buffering function (Huang et al., 2020). When teachers perceive strong support from school management, colleagues, and institutional structures, the negative impact of emotional labor (e.g., surface acting) can be partially mitigated. In contrast, under conditions of low organizational support, the psychological cost of emotional labor may be amplified. This moderating effect is in line with previous research, which suggests that adequate POS helps reduce teachers' stress and burnout (Rhoades and Eisenberger, 2002). For instance, a positive school climate and supportive interpersonal relationships have been shown to enhance teachers' professional efficacy while reducing emotional exhaustion and turnover intention (Richards et al., 2016). Although some studies have failed to detect a significant buffering effect of POS on the link between emotional labor and burnout (Anomneze et al., 2016), the majority of evidence supports POS as a critical contextual resource that can buffer the negative effects of job demands on burnout. For preschool teachers in particular, organizational recognition, resource provision, and emotional support play vital roles in helping them cope effectively with emotional labor demands. These organizational resources also enhance the utility of personal strengths such as mindfulness, ultimately safeguarding teachers' psychological wellbeing and reducing burnout.

While this study confirms the positive role of mindfulness in alleviating preschool teachers' burnout, we must recognize that mindfulness is not a “universal panacea” applicable to all workplace contexts. Indeed, recent critical literature has identified multiple limitations in organizational applications of mindfulness. First, when organizations instrumentalize mindfulness as a tool to enhance employee performance or emotion regulation rather than genuinely addressing wellbeing, it risks being co-opted as a covert control mechanism—encouraging employees to self-adjust to potentially unreasonable organizational demands rather than driving structural reforms (Lyddy et al., 2021). Moreover, commercially-driven mindfulness interventions often become divorced from their ethical foundations in Buddhist tradition, reduced to decontextualized psychological techniques that may reinforce performance-oriented goals rather than holistic employee development (Hyland, 2017). Additionally, the non-linear “surface acting-mindfulness” relationship revealed in our study suggests that highly mindful individuals performing sustained emotional labor may experience heightened discomfort due to acute awareness of internal conflicts, potentially accelerating self-regulatory resource depletion in certain contexts (Lyddy et al., 2021). These phenomena indicate that mindfulness doesn't operate in isolation; its efficacy depends fundamentally on organizational support systems and overall work environment quality. Without institutional support for emotional labor and substantive improvements in working conditions, standalone mindfulness training may prove ineffective or even counterproductive—creating a “mindfulness paradox” where greater awareness of organizational inequities exacerbates psychological distress (Purser, 2018).

## 5 Conclusion

This study examined the combined effects of surface acting and mindfulness on burnout among preschool teachers and further explored the mediating role of emotional empathy and the moderating role of POS. The results showed that individuals with high levels of mindfulness were better able to buffer the emotional depletion caused by surface acting, whereas those with low mindfulness were more susceptible to burnout under prolonged surface acting. In addition, emotional empathy played a mediating role in the pathway linking surface acting and mindfulness to burnout: surface acting undermined teachers' capacity for emotional empathy, while mindfulness enhanced it, thereby reducing levels of burnout. Moreover, POS moderated this relationship, such that teachers experienced lower levels of burnout in high-support environments and higher levels of burnout risk under conditions of low organizational.

At the theoretical level, this study expands the framework of emotional labor theory by demonstrating that the fit between different emotional regulation strategies—such as surface acting and mindfulness—may influence burnout differently depending on individual traits. It also deepens the application of mindfulness theory in the educational context by revealing that mindfulness not only directly reduces burnout but also indirectly enhances teachers' professional adaptability through improved emotional empathy. Furthermore, the study underscores the critical role of POS in mitigating burnout, confirming that external support systems can effectively alleviate preschool teachers' emotional burden, and offers new insights for school-level and policy-level teacher support strategies.

The findings of this study provide systematic empirical evidence and practical guidance for preschool education management and teacher psychological interventions. The non-linear interaction between surface acting and mindfulness on burnout (response surface analysis showing a significant congruence line slope a1 = 0.1232, *p* < 0.01) establishes a scientific basis for targeted interventions in educational institutions. Accordingly, we recommend implementing multi-level intervention strategies. At the individual level, differentiated mindfulness training programs should be developed: for high-risk “high surface acting-low mindfulness” teachers, basic mindfulness courses emphasizing emotional awareness should be designed to enhance emotion regulation; while advanced mindfulness training should be provided for “low surface acting-high mindfulness” teachers to sustain positive effects. Given the mediating role of emotional empathy [indirect effect 95%CI (0.312, 0.709)], empathy training modules incorporating techniques like role-playing should be integrated to foster authentic emotional connections with children. At the organizational level, since perceived organizational support (POS) significantly moderated the main effect (β = −0.292, *p* < 0.001), multi-tiered support systems should be established, including regular emotional care interviews by administrators (e.g., principal meetings), peer support groups, and improved career development pathways—aligning with Conservation of Resources theory's resource gain principle. Notably, the finding that burnout levels remained low in high surface acting-high mindfulness combinations when organizational support reached mean+1SD suggests potential for developing “emotional labor-mindfulness” synergistic training programs. Additionally, institutions should optimize emotional labor management by reducing compulsory emotional display rules and encouraging healthier regulation strategies like deep acting or mindful awareness. Building on the block variable analysis method employed in this study, we recommend developing teacher psychological monitoring systems to enable precise, data-driven interventions by regularly assessing surface acting-mindfulness combinations. These measures collectively form an integrated “individual-organization” dual-level protection system that both enhances teachers' personal psychological resources through mindfulness training and improves environmental resources through organizational support, fully embodying the job demands-resources balance concept of the JD-R model and providing a comprehensive, evidence-based solution for reducing preschool teachers' burnout risk.

While this study has achieved meaningful findings, several limitations warrant further investigation. First, the predominant reliance on self-report questionnaires introduces notable methodological constraints: social desirability bias may lead teachers to underreport surface acting behaviors (e.g., disguising fewer emotional expressions) while overestimating their mindfulness levels—particularly given the societal expectation for preschool teachers to maintain a “nurturing image”—and this reporting bias could substantially inflate perceived emotional awareness. Moreover, self-reports cannot capture unconscious emotional labor processes, whereas neuroscientific evidence indicates that prolonged emotion regulation often involves automated neurocognitive mechanisms (Gkintoni et al., 2025). Second, insufficient consideration of contextual factors poses critical limitations—the study sample may not fully represent regional disparities in China's early education system (e.g., urban-rural differences in teacher-student ratios and curricula), while culturally specific variables like distinctive Chinese “parent-teacher dynamics” (e.g., exceptionally high parental expectations) remain unexamined, thereby constraining the cultural interpretability of findings. Third, causal inference limitations manifest not only in the absence of experimental designs but also in longitudinal analytical strategies; although the three-wave design surpasses cross-sectional approaches, it cannot fully account for temporal confounders like abrupt policy changes affecting teachers' workload mid-semester. Finally, the study inadequately addresses cultural specificity in emotional labor: Hochschild's theory derives from Western service-sector research, whereas Chinese teachers may employ unique emotion regulation strategies (e.g., the Confucian “rounded exterior with principled core” approach) that existing measures fail to capture. Future research should: methodologically integrate physiological measures (e.g., heart rate variability monitoring) and objective indicators (e.g., teaching video coding); enhance cultural adaptability by developing indigenous emotional labor scales and examining interactions between traditional practices (e.g., Confucian self-cultivation) and modern mindfulness training; and employ intensive longitudinal designs (e.g., daily diary methods) or school-based natural experiments to strengthen causal claims. These advancements would markedly improve the ecological validity and cultural sensitivity of conclusions.

## Data Availability

The raw data supporting the conclusions of this article will be made available by the authors, without undue reservation.
